# Anti-malarial artesunate ameliorates atherosclerosis by modulating arterial inflammatory responses *via* inhibiting the NF-κB–NLRP3 inflammasome pathway

**DOI:** 10.3389/fphar.2023.1123700

**Published:** 2023-02-02

**Authors:** Yanyan Cen, Yalan Xiong, Rongxin Qin, Hui Tao, Qunfang Yang, Xichun Pan

**Affiliations:** Department of Pharmacology, College of Pharmacy, Army Medical University, Chongqing, China

**Keywords:** atherosclerosis, artesunate, atherogenic diet, inflammation, the NF-κB–NLRP3 inflammasome pathway

## Abstract

**Introduction:** Chronic inflammation plays a critical role in the pathogenesis of atherosclerosis (AS), and involves a complex interplay between blood components, macrophages, and arterial wall. Therefore, it is valuable in the development of targeted therapies to treat AS.

**Methods:** AS rat model was induced by atherogenic diet plus with lipopolysaccharide (LPS) and then treated by anti-malarial artesunate (Art), a succinate derivative of artemisinin. The arterial morphology was observed after Oil red O, hematoxylin—eosin, and Masson’s staining. The arterial protein level was detected by immunohistochemistry or immunofluorescence. The expression level of mRNA was determined by PCR array or real-time PCR.

**Results:** Herein, we showed that Art possessed a dose-dependently protective effect on AS rats. In detail, Art showed a comparable inhibitory effect on arterial plaque and serum lipids compared to those of rosuvastatin (RS), and further showed a better inhibition on arterial lipid deposition and arterial remodeling comprised of arterial wall thicken and vascular collagen deposition, than those of RS. The improvement of Art on AS rats was related to inhibit arterial macrophage recruitment, and inhibit nuclear factor κB (NF-κB)-related excessive arterial inflammatory responses. Critically, Art showed significant inhibition on the NLRP3 inflammasome activation in both arterial wall and arterial macrophages, by down-regulating the expression of NOD-like receptor thermal protein domain associated protein 3 (NLRP3) and apoptosis associated speckle-like protein containing CARD (ASC), leading to less production of the NLRP3 inflammasome—derived caspase-1, interleukin-1β (IL-1β), IL-18, and subsequent transforming growth factor β1 (TGF-β1) in AS rats.

**Conclusion:** We propose that Art is an anti-AS agent acts through modulating the arterial inflammatory responses *via* inhibiting the NF-κB – NLRP3 inflammasome pathway.

## Highlights

•Artesunate protects atherosclerotic rats induced by an atherogenic diet plus lipopolysaccharide.

•Artesunate modulates arterial inflammatory responses.

•Artesunate inhibits the NF-κB–NLRP3 inflammasome pathway.

## Introduction

Atherosclerosis (AS) is a fundamental cause of cardiovascular diseases (CVDs), accounting for about 50% of deaths worldwide (Libby et al., 2019). As a well-studied chronic disease, AS is characterized mainly by the formation and development of lipid and immune-cell containing plaques in the inner lining of the arteries (Libby et al., 2011; Schaftenaar et al., 2016). The pathogenesis of AS is complicated and involves numerous etiological factors, wherein lipid deposition and inflammatory response have been proposed to play key roles in the process of plaque development (Schaftenaar et al., 2016). It is quite attractive to unveil the regulatory mechanism of arterial lipid deposition and inflammatory response and develop promising therapy for AS treatment.

Arterial lipid deposition and inflammation form a complicated regulatory network during AS. Deposited lipids induce a series of arterial responses, including induction of inflammation, intimal accumulation of lipids, and foam cell formation, which play an essential role in AS, especially in the initial stage (Chistiakov et al., 2017). Arterial macrophages, differentiated from circulating monocytes, are responsible for clearance of deposited lipids to prevent cytotoxicity, tissue injury, inflammation, and metabolic disturbances (Chistiakov et al., 2016). Macrophages use pattern recognition receptors (PRRs) and subsequently initiate signaling cascades that lead to the transcription of pro-inflammatory cytokines and chemokines to manage lipid internalization (Grebe and Latz, 2013). However, excessively accumulated macrophages are also associated with chronic inflammation of vascular walls, which is strongly related to the initiation, growth, and rupture of arterial plaques, and vascular remodeling (Xu et al., 2019).

As a well-studied transcription factor, nuclear factor κB (NF-κB) is activated by pathogen-associated molecular patterns (PAMPs) or cytokines and then primes transcription of a series of genes encoding cytokines and chemokines in macrophages (Hu et al., 2021). In the past 3 decades, NF-κB has been regarded as the most critical player in AS because the genes it primed are involved in all phases of AS (Li et al., 2022). In addition, NF-κB also primes mRNA expression of NOD-like receptor thermal protein domain-associated protein 3 (NLRP3) and apoptosis-associated speckle-like protein containing CARD (ASC), which form NLRP3 inflammasome to cleave pro-caspase-1 into caspase-1, by which it converts pro-interleukin 1β (pro-IL-1β) and pro-IL-18 to mature forms (Baldrighi et al., 2017; Grebe et al., 2018). NLRP3 inflammasome has been considered a link between lipid metabolism and inflammation in AS (Li et al., 2016). As a critical pathogenic factor involved in all stages of AS, cholesterol crystals are one of the most potent activators of NLRP3 inflammasome to initiate and exacerbate AS *via* IL-1β and IL-18, which are well-known as the pro-atherogenic cytokines (Sharma and Kanneganti, 2021; Takahashi, 2022). In addition, reports indicated that NLRP3 expression was upregulated in the aorta of patients with coronary AS, and the aortic NLRP3 expression was correlated with the severity of disease (Afrasyab et al., 2016; Paramel Varghese et al., 2016). In various AS animal models, high NLRP3 levels were observed in monocytes and macrophages (Karasawa and Takahashi, 2017). Therefore, numerous reports have suggested that the NF-κB–NLRP3 inflammasome pathway is a possible target for future drug discoveries and clinical settings (Parsamanesh et al., 2019).

Previously, we reported that anti-malarial artesunate (Art) could protect AS in an apolipoprotein E-knockout (*ApoE* ^-/-^) mice model *via* inhibition of the release of chemokines including IL-8 and C–C motif chemokine ligand 2 (CCL2) (Jiang et al., 2016). In addition, recent reports also showed that Art possessed similar anti-AS activity in an *ApoE* ^-/-^ mice model (He et al., 2020; Wang et al., 2022). However, an ApoE-deficiency model which possesses the APO-dysfunction phenotype cannot fully account for the pathogenic factors, especially complicated inflammatory responses which have been regarded as the critical trigger during the AS process (Ross, 1999). Moreover, the detailed anti-AS mechanism of Art has not been well elucidated yet. In this study, we further evaluated the anti-AS activity of Art in a normal rat model induced by an atherogenic diet supplemented with lipopolysaccharide (LPS), which consisted of both a high-fat pathogenic factor and pro-inflammatory pathogenic factor that are widely used for accelerating the progression of AS (Wiesner et al., 2010; Yin et al., 2013), and the possible mechanism was also discussed from the perspective of inflammation controlled by the NF-κB–NLRP3 inflammasome pathway.

## Materials and methods

### Animal experiments

All animal experiments were performed in accordance with the National Guidelines for Animal Care and Use (NIH Publication No. 85–23, revised 1996) and approved by the Ethics Committee for Animal Experimentation of Army Medical University (License No. AMUWEC20181680). All surgeries and sacrifices were performed in a manner to minimize animal suffering.

Female Wistar rats (6–8 weeks old; 190 ± 20 g) were supplied by the Experimental Animal Center of our university and were housed under a pathogen-free condition (License No. SCXK-2017002). All rats were raised on a 12-h light/dark cycle with free access to water, and the temperature was 20°C ± 2°C with a humidity of 50 ± 5% following a 7-day acclimatization period. The inflammatory immune method was used for the animal model (Wiesner et al., 2010; Fu et al., 2017). A total of 70 rats were given 70,000 U/kg of vitamin D3 (i.p.; Sigma, St. Louis, MO, United States) three times in the first week, and 0.15 mg/kg of LPS (i.m.; Sigma) was given six times every week. These rats were fed with the atherogenic diet (1.25% cholesterol, 15% fat, and 0.5% cholic acid) for 8 weeks to induce acute AS. The rats used as a control (*n* = 10) were fed with a standard chow diet. At the end of the 8th week, 10 atherogenic diet rats were used for detecting serum biochemistry and observing histological changes of the aortic arch vessel. The rest were randomly divided into six groups (n = 10) for a further 8-week treatment. These rats were fed with the atherogenic diet containing normal saline (AS group), 4.5 mg/kg of Art (bid, H-bid group; qd, H-qd group; Guilin Pharmaceuticals, Guilin, Guangxi, China), 1.5 mg/kg of Art (bid, L-bid group; qd, L-qd group), or 4.8 mg/kg/day of rosuvastatin (RS group; Pfizer Inc., NY, United States). Art was dissolved in 5% sodium bicarbonate and was diluted in normal saline. All drug solutions were prepared at room temperature before use.

### Samplings

At the end of experiments, all rats were anesthetized with isoflurane and sacrificed. The abdominal aorta, full-length aorta, and peripheral blood were harvested, respectively. Serum samples were prepared using peripheral blood by centrifugation (3000 × g/min for 5 min) and then were used for detecting serum biochemistry. The thoracic aortas were quickly excised and collected for extracting total RNA or fixed with paraformaldehyde (4%; *m*/*v*) for 48 h and then embedded in paraffin for subsequent histological observations.

### Serum biochemistry and ELISA

The serum levels of total cholesterol (TC), triglyceride (TG), high-density lipoprotein cholesterol (HDL-C), and low-density lipoprotein cholesterol (LDL-C) were estimated using commercial kits (Boster, Wuhan, Hubei, China). The serum levels of caspase-1 and IL-1β were measured using corresponding enzyme-linked immunosorbent assay (ELISA) kits (Boster).

### Histological observations

Oil red O staining was performed to observe atherosclerotic lesions. Briefly, the heart and entire aorta were dissected free from the rats, and photographs were captured. The whole thoracic aortas from the same position of each sample were opened longitudinally and stained with Oil red O (Beyotime, Shanghai, China). The paraffin sections were stained by hematoxylin–eosin (HE; Boster, Wuhan, China), Masson’s trichrome (Boster), and Oil red O according to the user manuals. All section images were captured under an Olympus BX51 light microscope (Tokyo, Japan). The lesion-positive staining (%) values and the collagen-positive staining (%) values were analyzed using ImageJ (Schindelin et al., 2015).

### Immunohistochemistry

Immunohistochemistry (IHC) was performed using an IHC kit (Boster) according to the user manual. Briefly, the paraffin sections (*n* = 5) were probed with the primary antibodies (Cell Signaling, Beverly, MA, United States) against NF-κB p65 subunit (1:200 dilution), NLRP3 (1:200 dilution), ASC (1:200 dilution), collagen I and III (Col-I and Col-III; 1:200 dilution), IL-1β (1:200 dilution), IL-18 (1:200 dilution), and transforming growth factor β1 (TGF-β1; 1:200 dilution). The IHC images were captured under a light microscope (Olympus, Japan). Images were analyzed using the ImageJ packages (Schindelin et al., 2015).

### Immunofluorescence

Arterial immunofluorescence was performed (*n* = 5) as described previously (Zhuang et al., 2019). In summary, macrophages were probed by the F4/80 (Cell Signaling; 1:200 dilution) antibody, vascular smooth muscle cells were probed by the smooth muscle actin α (α-SMA; Cell Signaling; 1:200 dilution) antibody, and NLRP3 expression was probed by the NLRP3 antibody (Cell Signaling; 1:200 dilution). These proteins were then stained by the secondary antibody conjugated with Alexa Fluor 488 (Sigma; green; 1:400 dilution) or Alexa Fluor 555 (Sigma; red; 1:400 dilution) as indicated, followed by DAPI staining (Sigma; blue) for visualizing nuclear DNA. The images were captured under a Zeiss LSM780 confocal microscope (Jena, Germany) and were analyzed using ImageJ.

### PCR array

In accordance with the user manual, total RNA was extracted from the arterial samples from the control group, the AS group, and the Art treatment (H-bid) group (*n* = 3) using a TRIzol reagent (Takara, Dalian, Liaoning, China) and was reverse-transcribed afterward using PrimeScript™ RT Master Mix (Takara). Then, mRNA expression was detected using Cytokines and Chemokines PCR Array (Wcgene Biotech, Shanghai, China). All primers used are listed in Table 1. Data were analyzed with the comparative Ct method normalizing to *GAPDH* and *β-actin*. The heatmap was analyzed using an online server Heatmapper (www.heatmapper.ca/expression/). Rat peritoneal macrophages (RPMs) were isolated and cultured as described previously (Chen et al., 2020). RPMs grown in 12-well plates (5 × 10^5^ cells/well) were treated by LPS (10 ng/mL) plus soluble cholesterol (CHO; 100 μg/mL) with or without the presence of Art (20 μg/mL) for 1 h (*n* = 5). Similarly, total RNA of rat PMs was extracted and analyzed by Cytokines and Chemokines PCR Array as described previously.

**TABLE 1:** Primers used in this study.

Gene	GenBank accession no.	Forward primer (5’→3′)	Reverse primer (5’→3′)
*NLRP3*	NM_001191642	TGG​ATA​GGT​TTG​CTG​GGA​TA	CCA​CAT​CTT​AGT​CCT​GCC​AAT
*ASC*	AB053165	GCA​CAG​CCA​GAA​CAG​AAC​AT	TAC​AGA​GCA​TCC​AGC​AAA​CC
*TNF-α*	BC107671	ATG​AGC​ACG​GAA​AGC​ATG​ATC​CGA​G	CTC​GGA​TCA​TGC​TTT​CCG​TGC​TCA​T
*IL-1β*	BC091141	GCT​TCA​AAT​CTC​ACA​GCA​GCA​T	GCA​GGT​CGT​CAT​CAT​CCC​AC
*IL-6*	RATIL6	TTC​TTG​GGA​CTG​ATG​TTG​TTG	TAC​TGG​TCT​GTT​GTG​GGT​GGT
*IL-7*	AF367210	GAT​TGC​CCA​AAT​AAT​GAA​CC	TGT​GCC​GTC​TGA​AAC​TCT​TA
*IL-10*	RATIL10X	GCA​CTG​CTA​TGT​TGC​CTG​CTC​T	CCC​AAG​TAA​CCC​TTA​AAG​TCC​TG
*IL-12α*	NM_053390	CTG​AAG​ACC​ACG​GAC​GAC​AT	AGA​TGC​TAC​CAA​GGC​ACA​GG
*IL-17*	NM_001106897	ACC​TCA​ACC​GTT​CCA​CTT​CA	CAC​TTC​TCA​GGC​TCC​CTC​TTC
*IL-18*	AY258448	GCC​ATG​TCA​GAA​GAA​GGC​TCT	GCT​CCG​TAT​TAC​TGC​GGT​TGT
*IL-23*	AY055379	TTC​TCC​GTT​CCA​AGA​TCC​TTC	CTC​AGT​CAG​AGT​TGC​TGC​TCC
*IL-27*	XM_039101198	CTG​TTG​CTG​CTA​CCT​TTG​CTT	GTG​GAC​ATA​GCC​CTG​AAC​CTC
*TGF-β1*	BC076380	AAC​AAT​TCC​TGG​CGT​TAC​CTT	GCC​CTG​TAT​TCC​GTC​TCC​TT
*CCL-1*	NM_001191092	TGC​TGC​TTG​AAC​ACC​TTG​GA	TAG​CAG​GGC​TTC​ACC​TTC​TT
*CCL-2*	XM_039101198	CCC​CAA​TGT​TTC​CCT​GAC​CT	TGA​ATC​CTA​CAG​CCA​GCA​CC
*CCL-5*	NM_031116	GAC​ACC​ACT​CCC​TGC​TGC​TT	GTT​GAT​GTA​TTC​TTG​AAC​CCA​CTT
*CCL-11*	NM_019205	GCC​ACT​TCC​TTC​ACC​TCC​CA	TCA​GCG​TGC​ATC​TGT​TGT​TG
*CCL-17*	NM_057151	TGT​CAC​TTC​AGA​TGC​TGC​TCC	TTC​CCT​GGA​CAG​TCT​CAA​ACA
*CCL-21*	NM_001008513	CTA​CAA​CAT​TGT​CCG​AGG​CTA​C	CCT​TCC​TTT​CTT​CCC​AGA​CTT​A
*CXCL1*	NM_030845	CAG​TGG​CAG​GGA​TTC​ACT​TCA	GGG​ACA​CCC​TTT​AGC​ATC​TTT​T
*GM-CSF*	FGDFAD	TAT​ACA​AGC​AGG​GTC​TAC​GGG	ATG​AAA​TCC​TCA​AAG​GTG​GTG
*IFN-β*	NM_019127	TGC​CAT​TCA​AGT​GAT​GCT​CC	CAC​CCA​AGT​CAA​TCT​TTC​CTC​T
*β-actin*	NM_031144	CGT​AAA​GAC​CTC​TAT​GCC​AAC​A	CGG​ACT​CAT​CGT​ACT​CCT​GCT
*GAPDH*	NM_017008	CAA​GTT​CAA​CGG​CAC​AGT​CAA	GAT​CTC​GCT​CCT​GGA​AGA​TGG

### Real-time PCR

Total RNA extraction and reverse transcription were performed similarly to the aforementioned PCR array procedure. Subsequently, real-time PCR was performed using SsoAdvanced SYBR Green Supermix (Bio-Rad, Hercules, CA, United States) with primer pairs listed in Table 1. The mRNA levels (fold change) were calculated by normalizing to β-actin using the 2^−ΔΔCt^ method (Livak and Schmittgen, 2001).

### Statistical analysis

Data are presented as the mean ± standard deviation (S.D.). Differences were analyzed using Excel by the one-way ANOVA method for multiple comparisons (weight and feed intake) and by Student’s t*-*test method for comparison between individual groups. *p* < 0.05 was considered to be statistically significant.

## Results

### Art ameliorates AS in atherogenic diet–LPS-induced rats

**FIGURE 1:**
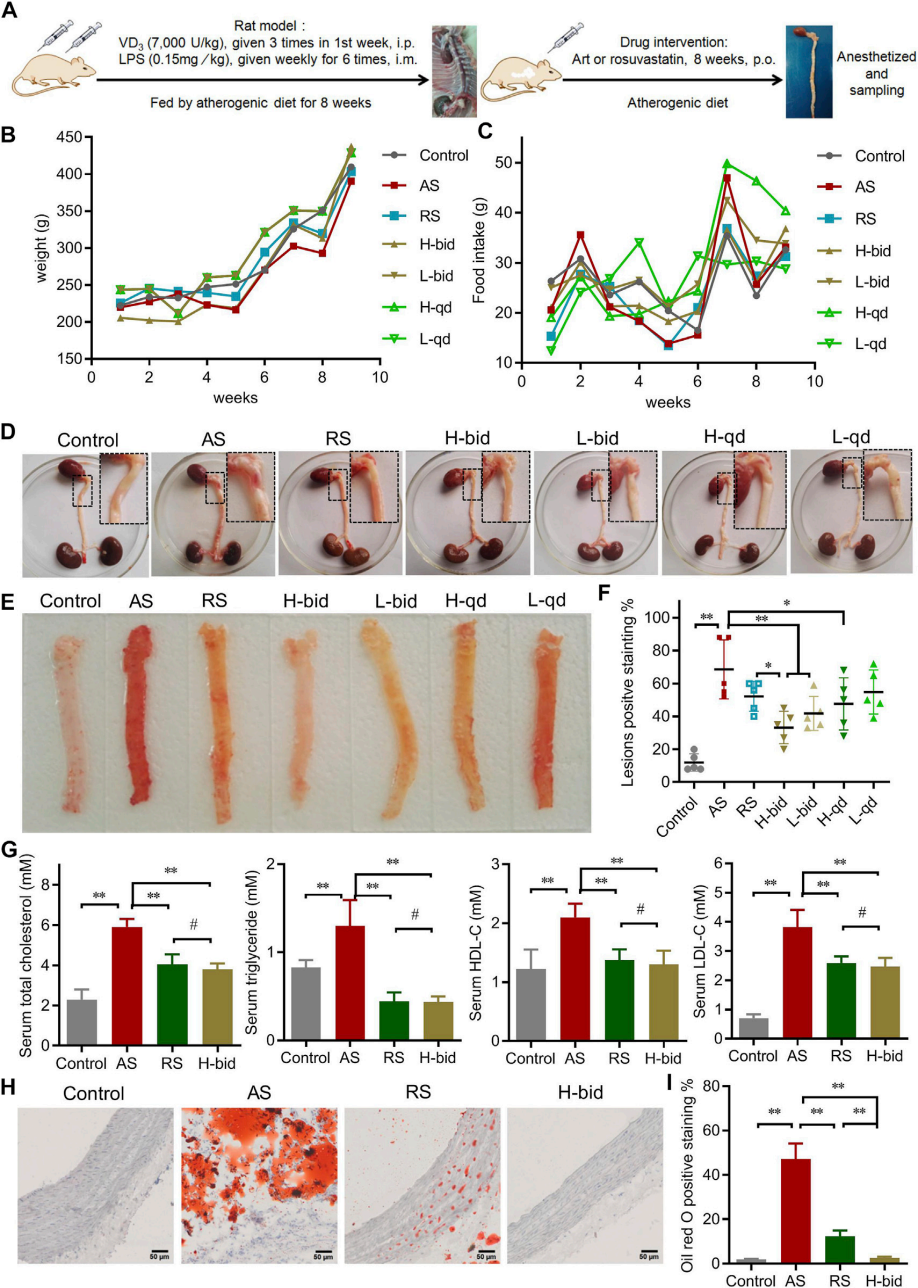
Art ameliorates AS in atherogenic diet–LPS-induced rats. **(A)** Procedure of the animal experiments. **(B)** Body weight of AS rats (*n* = 10). **(C)** Feed intake of AS rats (*n* = 10). **(D)** General observation of the heart and entire aorta. **(E–F)** Oil red O staining of aortas (*n* = 5). **(G)** Serum level of lipids (*n* = 5). **(H–I)** Oil red O staining of arterial sections. Paraffin sections were stained using Oil red O and then observed under a light microscope (Bar = 50 μm). The bar graph shows lesion-positive staining % (*n* = 5). ^*^ *p* < 0.05; ^**^ *p* < 0.01; ^#^ *p* > 0.05.

During the animal experiment (Figure 1A), no mortality was observed in any group. Compared to the control, the AS group, the RS-treated group, and all the Art-treated groups showed no significant influence on body weight and food intake of rats (Figures 1B,C). At the end of *in vivo* experiments, the heart and entire aorta were harvested, and the general observations showed that only the AS group demonstrated abnormal aortic morphology (Figure 1D).

Subsequently, Oil red O staining was applied to observe the atherosclerotic lesions of the aorta. Compared to the control group, the AS group possessed large and deep red lesions (75.2 ± 8.9%); however, those were markedly attenuated by Art or RS (Figures 1E,F). Among four groups of Art treatment, low-dose Art (1.5 mg/kg) showed lower positive staining (%) of 30.8 ± 10.6 (bid) and 65.5 ± 6.37% (qd), whereas high-dose Art (5.0 mg/kg) showed further lower positive staining (%) of 16.0 ± 5.8 (bid) and 26.2 ± 6.1 (qd). Critically, twice-daily Art groups, in spite of high or low dose, showed a significant difference compared to the AS group (*p* < 0.01). Moreover, the high-dose Art with twice-daily (H-bid) group showed lower positive staining (%) than the RS group (*p* < 0.05). These results demonstrate that Art attenuates the progression of AS in the rat model in a dose-dependent manner and can better protect the aorta AS than RS.

Hyperlipidemia is widely recognized as the most important risk factor for AS development (Libby et al., 2019). Therefore, the blood samples harvested at the end of *in vivo* experiments were used to detect serum lipids. The levels of serum Tch, TG, HDL-C, and LDL-C markedly increased in the AS group compared with those in the control group (Figure 1G). However, Art or RS treatment decreased all of these variables including Tch, TG, and LDL-C. In addition, the lipid-reducing capacity of Art showed no significant difference compared with that of RS. Therefore, our data suggest that Art possesses a strong antihyperlipidemic effect. Moreover, arterial paraffin sections were used to observe whether Art affected lipid deposition within plaques using Oil red O staining. Similar to the results of serum lipids, lipid deposition remarkably increased in the AS group but decreased by the use of RS or Art (Figures 1H,I). Interestingly, lipid deposition in the H-bid group was much lower than that in the RS group, which was very similar to the data from aortas (Figure 1F). Taken together, these findings indicate that Art shows similar inhibition on hyperlipidemia compared to RS and better inhibition on lipid deposition within plaques in the AS rat model.

### Art attenuates arterial remodeling in AS rats

**FIGURE 2:**
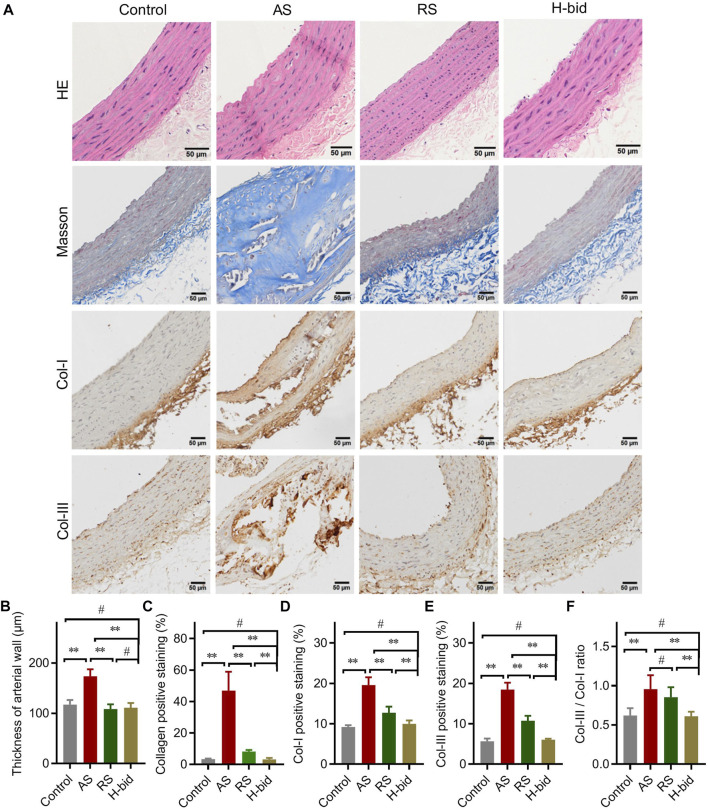
Art attenuates arterial remodeling in AS rats. **(A)** HE staining, Masson’s staining, and IHC staining of arterial sections. Paraffin sections were stained using HE, Masson’s trichrome, or were probed by antibodies against Col-I and Col-III using the IHC method and observed under a light microscope (Bar = 50 μm). **(B)** Thickness of the arterial wall. **(C)** Collagen-positive staining (%). **(D)** Col-I-positive staining (%). **(E)** Col-III-positive staining (%). **(F)** Col-III/Col-I ratio. *n* = 5; ^**^ *p* < 0.01; ^#^ *p* > 0.05.

Arterial remodeling, characterized predominantly by arterial wall thickening and vascular collagen deposition, is also a crucial alteration in AS (Evans et al., 2022). HE staining was performed to detect morphological changes in arteries. Compared to the control, arterial wall thickness of the AS group significantly increased, while that with RS or Art treatment was markedly declined (Figures 2A,B). The results indicate that Art shows inhibition on the arterial wall thickening similar to RS.

Collagen is a major component of the vascular intima and wall; hence, collagen deposition is considered to be along with the volume growth of these intimal lesions (Libby et al., 2019). Masson’s trichrome staining was applied to detect morphological changes in collagen deposition (Figure 2A). The AS group manifested massive collagen accumulation within the vascular smooth muscle layer, whereas treatment by RS or Art could alleviate collagen deposition induced by AS (Figures 2A,C). Consistently, IHC staining also indicated that the expression of Col-I and Col-III, the main members of interstitial collagen, was remarkably increased in the AS group but lessened significantly by RS or Art treatment (Figures 2A,D,E). Moreover, the Col-III/Col-I ratio, which indicates arterial stiffness and plaque stability, was also calculated herein. The Col-III/Col-I ratio was significantly increased in the AS group, whereas it was decreased by the treatment of only Art, not RS (Figure 2F). Critically, Art could restore collagen deposition and Col-III/Col-I ratio because there was no difference between the control group and the H-bid group (*p* > 0.05). In addition, Art showed better activity on decreasing AS-induced collagen deposition than the RS group (*p* < 0.01, as shown in Figures 2C–F). Taken together, the aforementioned findings suggest that Art can inhibit AS-induced arterial remodeling.

### Art attenuates arterial inflammation in AS rats

**FIGURE 3:**
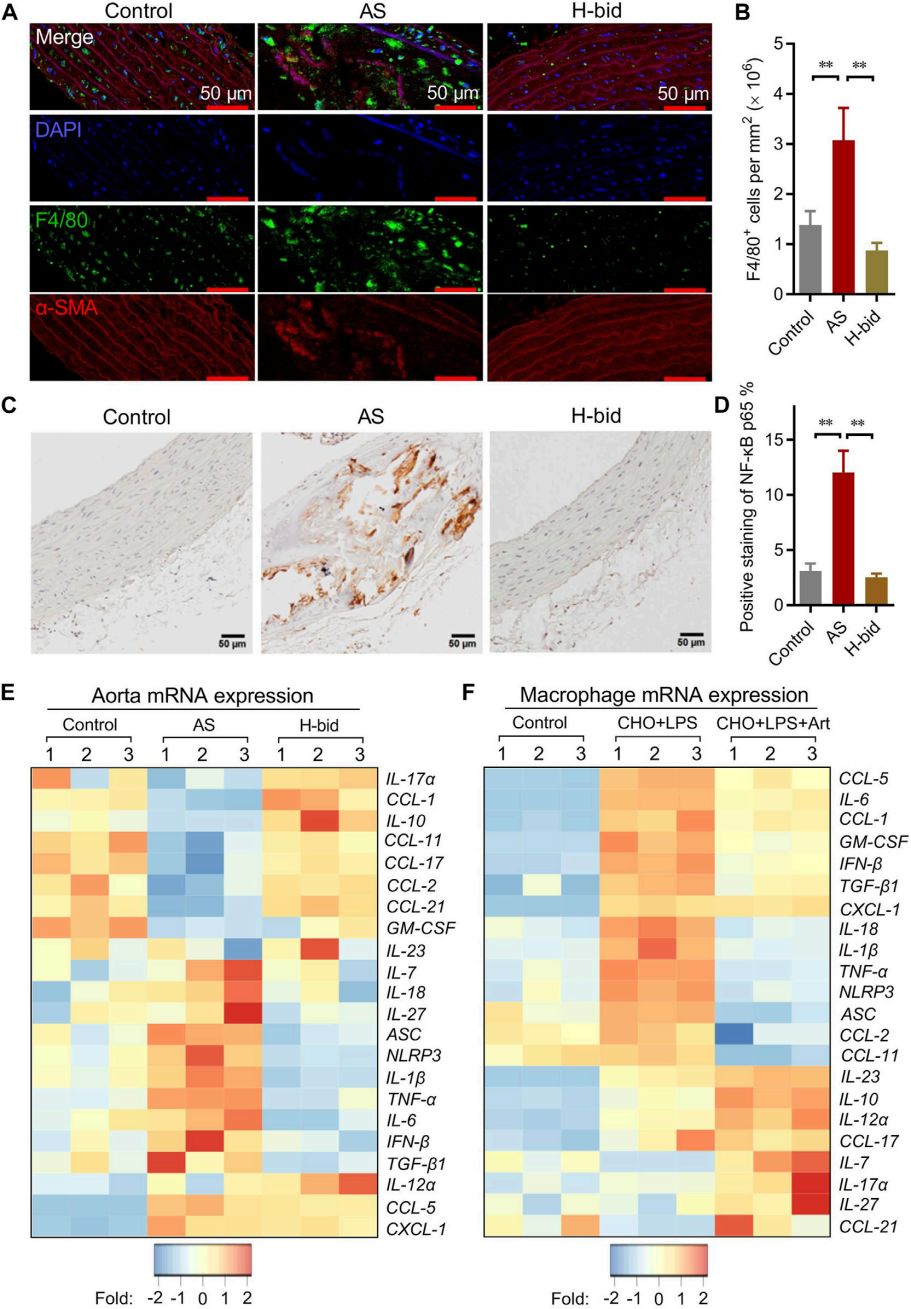
Art attenuates arterial inflammation in AS rats. **(A–B)** Immunofluorescence staining of arterial sections. Paraffin sections were probed with antibodies against F4/80 (green) or α-SMA (red), followed by DAPI (blue) staining. Confocal imaging was performed using a confocal microscope, and the F4/80^+^ cells (macrophages) were measured using ImageJ (Bar = 50 μm; *n* = 5). **(C–D)** IHC staining of arterial sections. Paraffin sections were probed by antibodies against the NF-κB p65 subunit using the IHC method and observed under a light microscope, and the percentage of NF-κB p65-positive staining was calculated using ImageJ (Bar = 50 μm; *n* = 5). **(E)** mRNA levels of a class of inflammatory cytokines and chemokines of aortas from atherosclerotic rats detected by PCR array (*n* = 3). **(F)** PCR array analysis of RPMs treated by water-soluble cholesterol (CHO; 100 μg/mL) plus LPS (10 ng/mL) with or without the presence of artesunate (Art; 20 μg/mL) for 1 h (*n* = 3). ^**^ *p* < 0.01.

Arterial macrophages, differentiated from circulating monocytes, are the main contributors responsible for lipid clearance, inflammatory responses, and arterial remodeling of AS (Libby et al., 2019). IF staining was applied to observe the influence of Art treatment on the recruitment of macrophages (F4/80^+^; green) by the arterial wall (α-SMA^+^; red) in AS rats. Our results showed that the macrophage number within plaques increased in the AS group while remarkably decreased by Art (Figures 3A,B). These findings indicate that Art inhibits, rather than increases, the recruitment of macrophages by arterial plaques in AS rats, which provides further evidence that Art inhibits arterial inflammation. Therefore, we detected whether Art affected the level of the NF-κB p65 subunit, which strictly regulated the inflammatory responses (Li et al., 2017), in aortas of AS rats using IHC staining. Not surprisingly, p65 was markedly upregulated in the AS group, especially within the plaques, but downregulated by Art (Figures 3C,D), indicating that Art shows an anti-inflammatory effect on the AS artery which is consistent with its inhibition of arterial macrophage recruitment.

Macrophage-derived pro-inflammatory cytokines and pro-inflammatory chemokines, operated by NF-κB, are responsible for arterial inflammation and plaque progression (Li et al., 2017). Therefore, the arterial mRNA levels of a class of cytokines and chemokines were detected *via* PCR array. As shown in the heatmap (Figure 3E), numerous genes were downregulated or were upregulated in the AS group, while the genes were restored in the H-bid group. Notably, we found several NF-κB-primed pro-inflammatory cytokines, including *IL-1β*, *IL-6*, *IL-18*, and *TNF-α* as well as *NLRP3* and *ASC*, which encoded the NLRP3 inflammasome-related proteins, were upregulated in the AS group, while those were downregulated by Art. To further confirm the findings from AS rats, we also performed similar PCR array experiments using RPMs. Similar to *in vivo* findings, we found that mRNA levels of *IL-1β*, *IL-6*, *IL-18*, *TNF-α*, *NLRP3*, and *ASC* were significantly upregulated by LPS plus CHO, whereas those were declined by Art (Figure 3F). Taken together, our data suggest that the protective effect of Art on AS rats is related to the decrease in inflammatory responses.

### Art inhibits arterial NLRP3 inflammasome activation in AS rats

**FIGURE 4:**
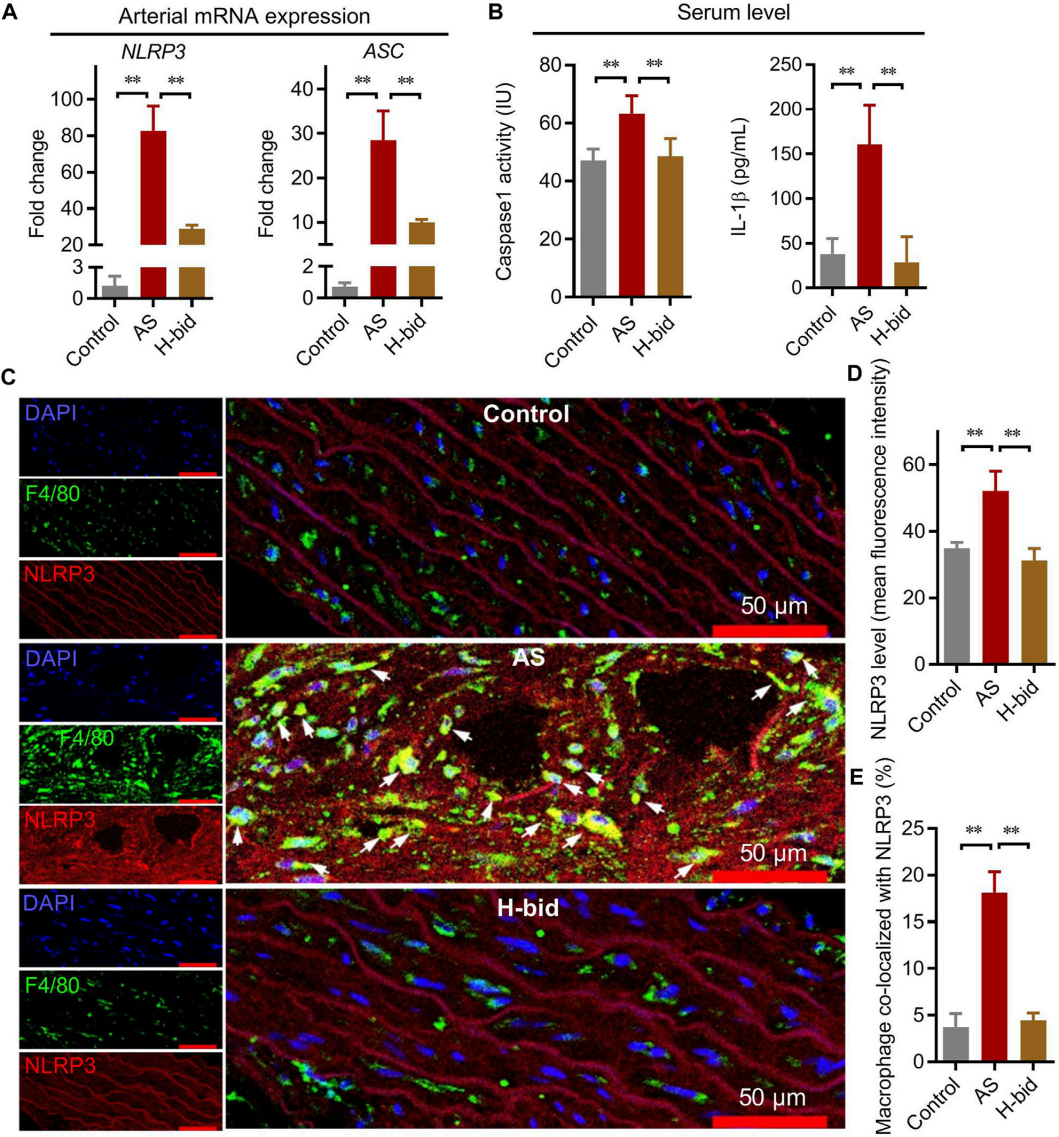
Art inhibits arterial NLRP3 inflammasome activation in AS rats. **(A)** mRNA expression of *NLRP3* and *ASC* in aortas from AS rats detected by real-time PCR (*n* = 4). **(B)** Serum levels of caspase-1 and IL-1β in AS rats detected by ELISA (*n* = 5). **(C)** Immunofluorescence staining of arterial sections. Experiments were performed as described in Figure 3A, using antibodies against F4/80 (green) or NLRP3 (red), followed by DAPI (blue) staining. The white arrows indicate the macrophages co-located with NLRP3. **(D–E)** Confocal images were analyzed by ImageJ; the arterial NLRP3 expression and colocalization of macrophages with NLRP3 were calculated, respectively (*n* = 5). ^*^ *p* < 0.05; ^**^ *p* < 0.01.

The NLRP3 inflammasome serves as a platform to activate caspase-1, thereby activating IL-1β and IL-18, which ultimately results in extensive collagen production and arterial remodeling (Grebe et al., 2018). Similar to mRNA expression of *IL-1β* and *IL-18*, that of *NLRP3* and *ASC* is also operated by NF-κB (Hoseini et al., 2018). Therefore, we further determined whether Art regulated mRNA expression of *NLRP3* and *ASC* in AS rats. It was shown that *NLRP3* and *ASC* mRNA expression was upregulated in the AS group but were downregulated by Art (Figure 4A). Moreover, the serum level of caspase-1 p10, which is an indicator of the activation of the NLRP3 inflammasome, was also determined. The results indicated that the serum level of caspase-1 p10 significantly increased in the AS group while decreased by Art (Figure 4B). Consistently, we found that the serum IL-1β level also remarkably increased in the AS group, whereas it decreased in the H-bid group (Figure 4B). Combining these findings, we can conclude that Art inhibits arterial NLRP3 inflammasome activation in AS rats.

However, whether Art inhibited NLRP3 inflammasome activation of macrophages or arterial walls was still unclear. Here, we observed NLRP3 expression with the indication of macrophages by F4/80 using immunofluorescence staining and found that NLRP3 was significantly upregulated in the arterial wall of the AS group, whereas it was downregulated by Art (Figures 4C,D). Critically, NLRP3 was significantly upregulated in arterial macrophages of the AS group, but it was downregulated by Art (Figures 4C,E). Collectively, aforementioned data suggest that Art inhibits NLRP3 inflammasome activation of both arterial wall and arterial macrophages in AS rats.

### Art inhibits the arterial NLRP3 inflammasome–TGF-β1 pathway in AS rats

**FIGURE 5:**
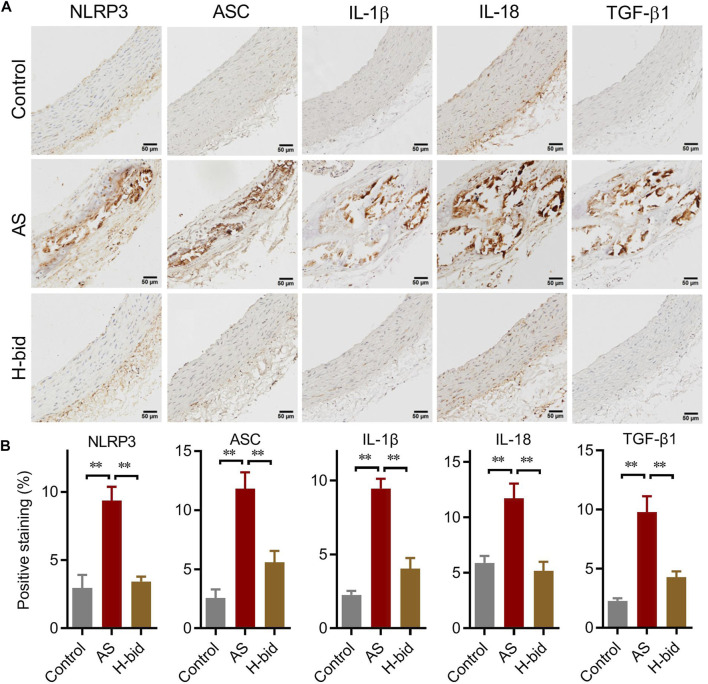
Art inhibits the arterial NLRP3 inflammasome–TGF-β1 pathway in AS rats. **(A)** IHC staining of arterial sections. IHC experiments were performed by using antibodies against NLRP3, ASC, IL-1β, IL-18, and TGF-β1 (Bar = 50 μm). **(B)** Percentages of positive staining (*n* = 5). ^**^ *p* < 0.01.

We further determined the main members and effectors of the NLRP3 inflammasome pathway in the arterial plaques of AS rats by IHC staining again. The results showed that the protein levels of two closely related inflammasome members, namely, NLRP3 and ASC, as well as two cytokines indicating inflammasome activation, namely, IL-1β and IL-18, were remarkably increased in the AS group, especially within arterial plaques; however, those were significantly decreased by Art (Figures 5A,B). Moreover, the expression of TGF-β1, a downstream AS effector of the NLRP3 inflammasome related to collagen production, was also upregulated in the AS group while downregulated by Art (Figures 5A,B). Thus, the protective effect of Art on AS rats is also related to its inhibition of the arterial NLRP3 inflammasome–TGF-β1 pathway.

## Discussion

In this study, we reported that Art showed comparable protective activity on AS rats induced by the atherogenic diet plus inflammatory stimulation compared to RS and further showed a better inhibition on arterial lipid deposition and arterial remodeling than RS. The protective effect of Art was to inhibit arterial macrophage recruitment and NF-κB-related excessive arterial inflammation, especially the NLRP3 inflammasome activation that leads to less production of caspase-1, IL-1β, IL-18, and subsequent TGF-β1. In conclusion, Art protects AS rats by modulating the arterial inflammatory responses *via* inhibition of the NF-κB–NLRP3 inflammasome pathway.

Since R. Ross raised the theory that “AS is an inflammatory disease” in 1999, a consensus of the pathogenesis of AS has been reached that inflammation plays a critical role and participates in the effects of many other risk factors for the development and complications of AS (Ross, 1999; Libby et al., 2011). It is well studied that some pathogens like *Chlamydia pneumoniae*, *Helicobacter pylori*, and cytomegalovirus are associated with AS, and the acute rise of C-reactive protein (CRP), which indicates infection, shows a strong positive correlation with AS (Stone and Kazil, 2014). Except these infectious factors, common CVDs, such as hypertension, hyperlipidemia, and diabetes, can stimulate the production of various inflammatory factors and thus affect the development of AS (Libby et al., 2011). Nowadays, the principles of immune response in experimental murine AS have been well addressed, and the efficacy of anti-inflammatory therapy in human AS has been confirmed in the clinical trials (Wolf and Ley, 2019). Collectively, inflammation has been validated as a critical mechanism in the pathogenesis of AS, and anti-inflammatory therapy has shown excellent clinical application value.

However, inflammation is indispensable in the pathological process of AS, responsible for waste clearance, vascular metabolic homeostasis, immune response, and repair of impaired vessels (Rosenfeld, 2013; Taleb, 2016). Therefore, researchers have to confront a contradictory challenge of finding a drug with a moderate anti-inflammatory effect while preserving the immune function of immune cells. During the complicated immunity and inflammation of AS, macrophage plays a central role by contributing to the maintenance of local inflammatory response, propagation of plaque development, recruitment of immune cells, and promotion of thrombosis (Barrett, 2020). In detail, macrophages are vital for the clearance of lipids and apoptotic cells; cytokines and chemokines released from macrophages are associated with subsequent recruitment of immune cells, maintenance of the vascular microenvironment, and matrix degradation which may lead to plaque instability (Taleb, 2016). Taking these into consideration, antimalarial Art, which possesses a moderate anti-inflammatory effect and immunomodulatory effect reported by our laboratory previously (Jiang et al., 2016; Kuang et al., 2018), comes into our sight.

Previously, we have showed that Art conferred a protective effect on the *ApoE* ^-/-^ mice AS model by declining release of IL-8 and CCL2 (Jiang et al., 2016). Reports from other groups have reported similar protective activity of Art in the *ApoE* ^-/-^ mice model in recent years (He et al., 2020; Wang et al., 2022). However, a typical *ApoE* ^-/-^ model confers a significant increase in the TC level and spontaneous AS lesions, due to the increased serum level of very low-density lipoprotein (VLDL) caused by ApoE-deficiency (Veseli et al., 2017). To our knowledge, ApoE function is normal in most human patients with AS. Meanwhile, the ApoE-deficiency model cannot fully account for other pathogenic factors, especially inflammation that lies in the center of AS. Therefore, we employed an AS model using normal rats fed with the atherogenic diet plus LPS, which comprised both the high-fat pathogenic factor and pro-inflammatory pathogenic factor. In this study, Art was administered at an antimalarial dose and showed a good protective effect on AS rats because the occurrence of AS lesions and the serum lipid level were almost completely restored by Art (as shown in Figure 1). Dramatically, Art not only showed comparable activity against AS similar to a potent statin drug, RS, but also showed a stronger inhibition on arterial lipid deposition and vascular remodeling than RS. Moreover, our previous report also indicated that Art showed a synergistic effect with RS against the *ApoE* ^-/-^ mice model (Jiang et al., 2016). These findings suggest that Art monotherapy and Art–RS combination therapy have a certain potential for future clinical application.

Considering that Art was more effective than RS in inhibiting lipid deposition and vascular remodeling and had a synergistic effect with RS, we proposed that Art might function through a different mechanism compared to RS. In combination with our previous reports, indicating that Art was an inflammatory modulator in infectious diseases, and the consensus that inflammation played a central role in AS, we began to explore the mechanism from the perspective of inflammation. Art showed a moderate inhibition of arterial recruitment of macrophages. As the dominant producer of cytokines and chemokines, excessively infiltrated macrophages within arteries would probably lead to hyperinflammatory and hyperimmune states, which might result in an increased plaque number in the initiation stage and a higher risk of plaque rupture in the pathological process of AS (Colin et al., 2014). Therefore, this feature of Art might be the basis for its anti-AS activity. In contrast, it is not surprising that hydroxychloroquine, which possesses a strong anti-inflammatory activity, has failed in the treatment of AS, unless AS is accelerated in systemic lupus erythematosus and chronic kidney disease, because patients with autoimmune disorders are in the hyperinflammatory stage which requires treatment with a potent anti-inflammatory agent (Shukla et al., 2015; Floris et al., 2018).

NF-κB is considered the most critical transcription factor, and it controls the transcription of cytokines, chemokines, matrix metalloproteinases, and adhesion factors, which contribute to the pathogenesis of AS (Evans et al., 2022; Li et al., 2022). NF-κB-targeted therapy using inhibitory NF-κB decoy oligodeoxynucleotide in a LPS/high-fat diet-induced AS mice model, which was very similar to our model, has exhibited protective activity on AS mice (Kim et al., 2010). In this study, we showed very consistent findings in a similar rat model using Art, confirming that NF-κB-targeted therapy is a promising strategy for AS treatment. Moreover, NF-κB-targeted therapy reduced pro-inflammatory cytokines, TNF-α and IL-1β, and inflammatory markers, vascular adhesion molecule (VCAM)-1 and intercellular adhesion molecule (ICAM)-1, in AS mice (Lee et al., 2013). In more detail, we showed the alterations of the inflammatory gene profile regulated by NF-κB in AS arteries and further exhibited changes of those in macrophages treated with CHO plus LPS. Art treatment attenuated AS-induced inflammation by declining production of pro-inflammatory cytokines, including TNF-α, IL-1β, IL-6, and IL-18.

Particularly, we found that mRNA expression of *IL-1β*, *IL-18*, and *TGF-β1* was downregulated by Art compared to the AS group *in vivo* and *in vitro*. Although the transcription process of these genes was operated by NF-κB, their protein level was tightly regulated by the activation of NLRP3 inflammasome (Baldrighi et al., 2017; Grebe et al., 2018). In brief, NLRP3 and ASC, the two core members of the NLRP3 inflammasome which were also primed by the NF-κB signaling, form an intact inflammasome with pro-caspase-1, and then mature caspase-1 cleaves pro-IL-1β and pro-IL-18 into IL-1β and IL-18, which in turn initiate TGF-β1 release and Smad activation that culminate in the production of collagen (Mangan et al., 2018). As suggested in recent studies, NLRP3 inflammasome is the main PRR for sensing cholesterol crystals in AS lesions and is essential for vascular inflammation and the progression of AS (Karasawa and Takahashi, 2017). Therefore, the NLRP3 inflammasome is widely recognized as the relevant target for AS treatment, and several NLRP3-targeted studies have confirmed the validity of this viewpoint (Kong et al., 2016; Parsamanesh et al., 2019; Zhang et al., 2019). Here, Art treatment indeed inhibits activation of the NLRP3 inflammasome, thereby reducing TGF-β1 production and decreasing arterial collagen deposition, ultimately leading to better protective activity in AS rats than RS.

Conclusively, Art possesses a good protective activity in AS rats and functions through inhibiting the NF-κB–NLRP3 inflammasome pathway. However, this study still has certain limitations. Although the protective effect of Art is well addressed, the relationship between the activity and the NF-κB–NLRP3 inflammasome pathway needs to be further investigated in NF-κB/NLRP3-deficiency animal models. Moreover, the influence of Art on arterial remodeling in AS rats should be observed more elaborately, such as the relationship between the time point of intervention and arterial remodeling. However, in consideration of the safety records of Art in treating malaria in the past 2 decade and its effectiveness in AS rats herein and *ApoE* ^-/-^ mice reported previously, we propose Art to be a promising agent for AS treatment.

## Data Availability

The original contributions presented in the study are included in the article/Supplementary Material; further inquiries can be directed to the corresponding author.
